# Mining prognostic markers of Asian hepatocellular carcinoma patients based on the apoptosis-related genes

**DOI:** 10.1186/s12885-021-07886-6

**Published:** 2021-02-18

**Authors:** Junbin Yan, Jielu Cao, Zhiyun Chen

**Affiliations:** The Second Central Laboratory, Key Lab of Integrative Chinese and Western medicine for the Diagnosis and Treatment of Circulatory Diseases of Zhejiang Province, The First Affiliated Hospital of Zhejiang Chinese Medical University, Hangzhou, 310006 China

**Keywords:** Asia, Hepatocellular carcinoma, Apoptosis-related genes(Args), Prognostic model, Risk score

## Abstract

**Background:**

Apoptosis-related genes(Args)play an essential role in the occurrence and progression of hepatocellular carcinoma(HCC). However, few studies have focused on the prognostic significance of Args in HCC. In the study, we aim to explore an efficient prognostic model of Asian HCC patients based on the Args.

**Methods:**

We downloaded mRNA expression profiles and corresponding clinical data of Asian HCC patients from The Cancer Genome Atlas (TCGA) and International Cancer Genome Consortium (ICGC) databases. The Args were collected from Deathbase, a database related to cell death, combined with the research results of GeneCards、National Center for Biotechnology Information (NCBI) databases and a lot of literature. We used Wilcoxon-test and univariate Cox analysis to screen the differential expressed genes (DEGs) and the prognostic related genes (PRGs) of HCC. The intersection genes of DEGs and PGGs were seen as crucial Args of HCC. The prognostic model of Asian HCC patients was constructed by least absolute shrinkage and selection operator (lasso)- proportional hazards model (Cox) regression analysis. Kaplan-Meier curve, Principal Component Analysis (PCA) analysis, t-distributed Stochastic Neighbor Embedding (t-SNE) analysis, risk score curve, receiver operating characteristic (ROC) curve, and the HCC data of ICGC database and the data of Asian HCC patients of Kaplan-Meier plotter database were used to verify the model.

**Results:**

A total of 20 of 56 Args were differentially expressed between HCC and adjacent normal tissues (*p* < 0.05). Univariate Cox regression analysis showed that 10 of 56 Args were associated with survival time and survival status of HCC patients (*p* < 0.05). There are seven overlapping genes of these 20 and 10 genes, including BAK1, BAX, BNIP3, CRADD, CSE1L, FAS, and SH3GLB1. Through Lasso-Cox analysis, an HCC prognostic model composed of BAK1, BNIP3, CSE1L, and FAS was constructed. Kaplan-Meier curve, PCA, t-SNE analysis, risk score curve, ROC curve, and secondary verification of ICGC database and Kaplan-Meier plotter database all support the reliability of the model.

**Conclusions:**

Lasso-Cox regression analysis identified a 4-gene prognostic model, which integrates clinical and gene expression and has a good effect. The expression of Args is related to the prognosis of HCC patients, but the specific mechanism remains to be further verified.

**Supplementary Information:**

The online version contains supplementary material available at 10.1186/s12885-021-07886-6.

## Background

At present, liver cancer has become one of the common causes of malignancy-related death globally [1], and its incidence is increasing year by year [2]. The World Health Organization (WHO) predicts that more than 1 million people will die of liver cancer by 2030 [3]. The incidence of liver cancer has remained high in Asia, as well [4]. HCC, as the main histological subtype of liver cancer, accounts for 90% of primary liver cancer. HCC is usually caused by various risk factors, including hepatitis B virus, hepatitis C virus, fatty liver, alcohol-related cirrhosis, smoking, obesity, different dietary exposures, etc. [5, 6]. Because HCC is a highly heterogeneous disease [7, 8], it is difficult to predict the prognosis. HCC is highly prevalent in Asia, and it is challenging to predict prognosis. Therefore, it is necessary to develop a useful prognostic model for Asian HCC patients.

Apoptosis is a way of programmed cell death related to the changes in cell morphology and structure [9]. Apoptosis mainly includes exogenous pathways caused by death receptors on the cell surface and endogenous apoptotic pathways caused by drugs, chemicals, endoplasmic reticulum stress, perforin, and granzyme [10]. Effector caspases activated by apoptosis signals will destroy the inhibitor of apoptosis, start the activity of caspase-activated deoxyribonuclease (CAD), and then destroy the structure of DNA, inhibit the activity of proteins regulating cell structure, destroy cell structure, transform cells into apoptotic bodies [11]. Apoptosis is closely related to HCC, the defect of apoptosis-inducing pathways will lead to the abnormal proliferation of tumor cells, and the resistance of cells to apoptosis will also increase the ability of tumor cells to evade immune system surveillance [12]. Args are closely related to the progression of HCC. Therefore, we intended to use the data of Asian HCC patients in TCGA database to construct a poly-genes prognostic model of Args and verify it by the data of ICGC database and Kaplan-Meier plotter database.

**Fig. 1:**
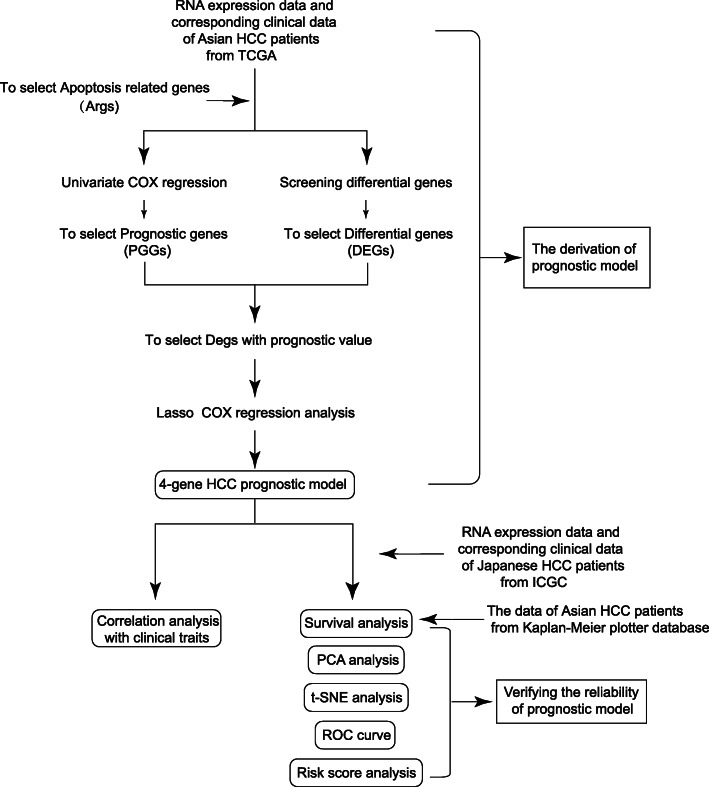
Flow chart of data collection and analysis

In the study, we downloaded the mRNA expression profiles and corresponding clinical data of Asian HCC patients from TCGA database. Japanese HCC patients’ data from ICGC and Asian HCC patients’ data from Kaplan-Meier plotter database were used for validation. Through Wilcoxon-test, we found the Args which were differentially expressed between HCC and adjacent normal tissues. Univariate Cox regression analysis showed some Args were associated with the survival time and survival status of HCC patients. By taking the intersection, we found that some Args were not only differentially expressed but also correlated with the prognosis of HCC. On this basis, we used Lasso-Cox analysis to mine a HCC prognostic model. Next, we used Kaplan-Meier curve, PCA, t-SNE analysis, risk score curve, ROC curve, and the HCC data from ICGC database and Kaplan-Meier plotter database to verify the prognosis effect of the model. The overall analysis flow was shown in Fig. 1.

## Methods

### Data preparation and processing

As of August 31, 2020, we downloaded the RNA-seq data and corresponding clinical characteristics of 158 Asian HCC patients from TCGA database (https://portal.gdc.cancer.gov/), including 6 normal paracancerous samples and 160 tumor samples, and the RNA-seq data and corresponding clinical information of 231 HCC patients from ICGC database (https://dcc.icgc/). The data of the 231 HCC patients downloaded from ICGC database are mainly from Japan [13]. The data of 155 Asian HCC patients from Kaplan-Meier plotter database (http://kmplot.com/analysis/) was selected as well [14]. To obtain the gene expression and clinical traits, we used R package limma [15] to process and normalize the data. We also downloaded Args from Deathbase dataset (http://deathbase.org/) and referred to the research results of GeneCards database (https://www.genecards.org), NCBI database (https://www.ncbi.nlm.nih.gov/), literature.

### Identification of prognostic apoptosis-related DEGs in the TCGA dataset

The expression of Args was screened from the TCGA expression matrix. The average value of repetitive genes was taken; the abnormal values were deleted. Through Wilcoxon-test, the genes with *p* < 0.05 were selected as DEGs. We used Univariate Cox analysis to combine the expression of Args with survival time and survival status of HCC patients to screen out PRGs. The screening condition was *p* < 0.05. The DEGs and PRGs were intersected to select the Args, which were not only differentially expressed between HCC tissues and adjacent normal tissues but also related to the prognosis of HCC. The protein-protein interaction network was drawn by STRING database (version11.0) (https://string-db.org/) to clarify the interaction of the proteins, and the expression correlation coefficient diagram was drawn by R package igraph to show the expression relationship of the genes. R package pheatmap was used to illustrate the expression heatmap to show the expression of the intersect ARGs. The correlation filtering threshold is 0.2. R package survival was used to clarify the relationship between the genes and the prognosis of HCC patients. The screening condition was *p* < 0.05, as well.

### Construction an Args related prognostic model of Asian HCC patients

The Lasso-penalized Cox regression analysis was applied to construct a prognostic model [16]. We used R package glmnet and lasso algorithm to contract variables, screen the genes that are positively related to the prognosis of HCC, and delete the genes with high correlation, to effectively avoid over-fitting of the prognostic model. The risk score of patients was calculated according to the expression of each gene and its corresponding regression coefficient. The formula was: Risk score = e^sum (expression of each gene * corresponding coefficient)^. Next, the HCC patients of TCGA and ICGC were divided into the high-risk and the low-risk groups according to the median cut-off risk score of the TCGA dataset.

### Validation of the prognostic model

According to the gene expression of the prognostic model, R package Stats and Rtsne were used for PCA analysis and t-SNE analysis to reduce the dimensionality of data, to explore the distribution of the high-risk and low-risk samples. R package survival and survminer were used to draw the Kaplan-Meier curve to show the overall survival (OS) of different groups. We also illustrated the risk curves to show the relationship between OS and risk scores of patients in two groups. Univariate and multivariate prognostic analysis, ROC curve drawn by R package timeROC were used to determine whether the model can be regarded as a useful biomarker relating to the prognosis of Asian HCC patients. To rule out accidental error, we used the expression data of Japanese HCC patients downloaded from ICGC database for secondary detection. We also used the data of 155 Asian HCC patients of Kaplan-Meier plotter database to draw the Kaplan-Meier curve to show the correlation between the expression of BAK1, BNIP3, CSE1L, FAS and overall survival (OS) to verify the reality of this prognostic model further.

### Correlation analysis with clinical traits

At first, we classified the clinical characteristics of HCC patients. Next, the model gene expression and risk score were compared with the clinical traits. T-test was used to determine whether the gene expression and risk score were associated with the clinical characteristics of HCC patients. The screening condition was *p* < 0.05. And the relationship between OS and clinical traits (Stage, Gender, Age) were displayed by the Kaplan-Meier curves.

### Statistical analysis

Wilcoxon-test was used to screen DEGs. Kaplan-Meier analysis was used to compare the differences in OS among different groups. Cox regression analysis of univariate and multivariate variables was performed to determine the independent prognostic factors. All statistical analyses were carried out with R software (3.6.2). *P* < 0.05 is considered to be statistically significant. All the data were calculated to the nearest ten-thousandth.

## Results

### The results of data preparation and processing

A total of 158 Asian HCC patients from the TCGA-LIHC dataset, 231 Japanese HCC patients from the ICGC (LIRI-JP) dataset, and 155 Asian HCC patients from Kaplan-Meier plotter database were selected. We synthesized the data of Deathbase dataset, GeneCards dataset, NCBI database, and related literature. A total of 56 Args were selected (Table 1).

**Table 1:** The Information of ARGs

Symbol	Description	Reference
ANXA1	Annexin A1	[11, 17]
APAF1	Apoptotic protease-activating factor 1	[18]
AVEN	Cell death regulator Aven	[19]
BAD	Bcl2 antagonist of cell death	[20]
BAK1	Bcl-2 homologous antagonist/killer	[21]
BAX	Apoptosis regulator BAX	[22]
BCL2	Apoptosis regulator Bcl-2	[23, 24]
BCL2A1	Bcl-2-related protein A1	[25, 26]
BCL2L1	Apoptosis regulator Bcl-X	[27]
BCL2L10	Apoptosis regulator Bcl-B	[28]
BCL2L11	Bcl-2-like protein 11	[29]
BCL2L12	Bcl-2-like protein 12	[30]
BCL2L14	Bcl-2-like protein 14	[31]
BCL2L2	Bcl-2-Like Protein 2	[32]
BID	BH3-interacting domain death agonist	[33, 34]
BIK	Bcl-2-interacting killer	[35, 36]
BIRC2	Baculoviral IAP repeat-containing protein 2	[37, 38]
BIRC3	Baculoviral IAP repeat-containing protein 3	[38, 39]
BMF	Bcl-2-modifying factor	[40]
BNIP3	BCL2/adenovirus E1B 19 kDa protein-interacting protein 3	[41]
BNIP3L	BCL2/adenovirus E1B 19 kDa protein-interacting protein 3-like	[42, 43]
CALR	Calreticulin Precursor	[44]
CASP10	Caspase-10 Precursor	[45]
CASP3	Caspase-3 Precursor	[46]
CASP6	Caspase-6 Precursor	[47]
CASP7	Caspase-7 Precursor	[48]
CASP8	Caspase-8 Precursor	[49]
CASP9	Caspase-9 Precursor	[50]
CFLAR	CASP8 and FADD-like apoptosis regulator Precursor	[51]
CRADD	Death domain-containing protein CRADD	[52, 53]
CSE1L	Exportin-2	[54]
CYCS	Cytochrome c	[55]
DIABLO	Diablo homolog, mitochondrial Precursor	[56]
FADD	Protein FADD	[57, 58]
FAS	Tumor necrosis factor receptor superfamily member 6 Precursor	[59]
FASL	Tumor necrosis factor ligand superfamily member 6	[60, 61]
HRK	Activator of apoptosis harakiri	[62]
LRP1	Prolow-density lipoprotein receptor-related protein 1 Precursor	[63]
MCL1	Induced myeloid leukemia cell differentiation protein Mcl-1	[64]
MOAP1	Modulator of apoptosis 1	[65]
NOXA	Phorbol-12-myristate-13-acetate-induced protein 1	[66]
PHAP	Acidic leucine-rich nuclear phosphoprotein 32 family member A	[67]
PUMA	Bcl-2-binding component 3	[68]
RIPK1	Receptor-interacting serine/threonine-protein kinase 1	[69]
SH3GLB1	Endophilin-B1	[70]
TNF	Tumor necrosis factor Precursor	[71]
TNFRSF1A	Tumor necrosis factor receptor superfamily member 1A Precursor	[72]
TNFRSF1B	Tumor necrosis factor receptor superfamily member 1B Precursor	[73]
TNFSF10	Tumor necrosis factor ligand superfamily member 10	[74]
TP53	Cellular tumor antigen p53	[75]
TRADD	Tumor necrosis factor receptor type 1-associated DEATH domain protein	[76]
TNFRSF10A	Tumor necrosis factor receptor superfamily member 10A Precursor	[77]
TNFRSF10B	Tumor necrosis factor receptor superfamily member 10B Precursor	[77]
TNFRSF10C	Tumor necrosis factor receptor superfamily member 10C Precursor	[78]
TNFRSF10D	Tumor necrosis factor receptor superfamily member 10D Precursor	[78]
XIAP	Baculoviral IAP repeat-containing protein 4	[79]

### The results of the screening of prognostic apoptosis-related DEGs

**Fig. 2:**
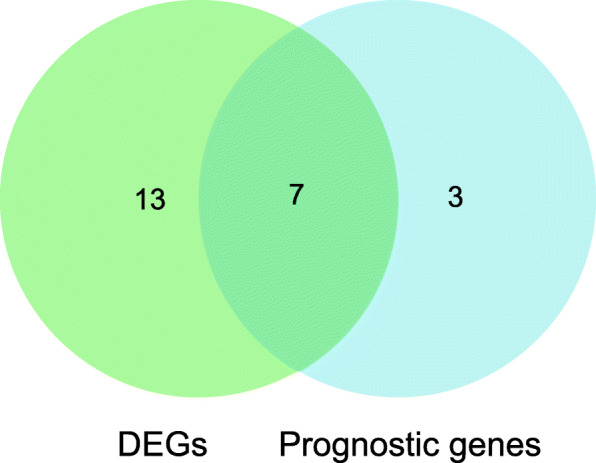
Venn diagram showing the intersect genes of differential genes (DEGs) and prognostic gens (PGGs)

**Fig. 3:**
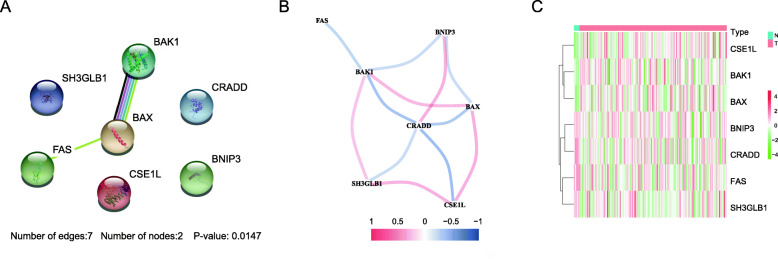
The analysis of 7 intersect ARGs. **a** The PPI network. **b** The expression correlation network. **c** The expression heatmap

**Fig. 4:**
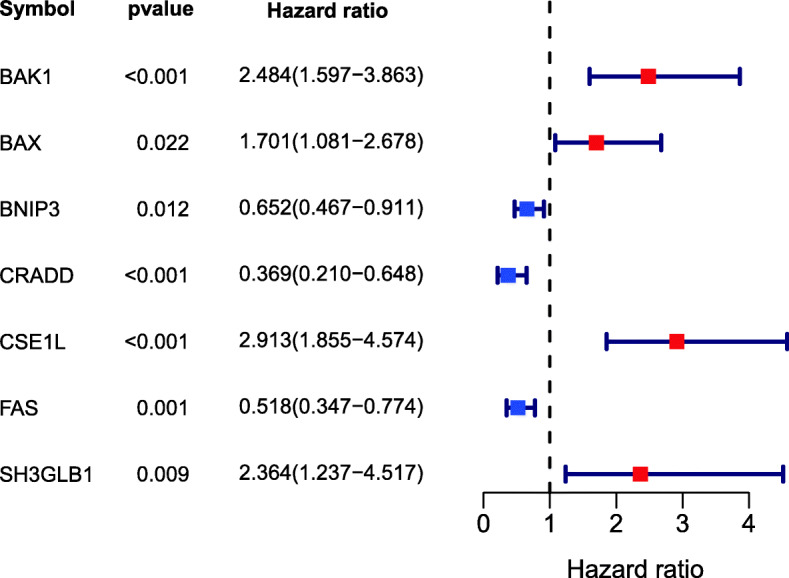
Forest plot showing the results of the univariate Cox regression analysis between gene expression and OS (overall survival)

We found that a total of 20 Args were differentially expressed between HCC tissues and adjacent normal tissues (*p* < 0.05) (Table 2). Through univariate Cox regression analysis, we found that a total of 10 Args genes were related to the prognosis of HCC patients (*p* < 0.05) (Table 3). The intersection of the DEGs and PGRs was taken, seven apoptosis-related prognostic DEGs were screened out (Fig. 2). There are BAK1, BAX, BNIP3, CRADD, CSE1L, FAS, and SH3GLB1. Using the confirmed protein interaction relationship of String database [80], a PPI protein interaction network was constructed (Fig. 3a). The results showed that BAK1, BAX have the most edges in the network. The R package igraph was used to draw the correlation network according to the coefficient of the expression of the above gene. Figure 3b showed that according to the expression, BAK1, BAX, and CRADD might be the crucial genes of the network. Figure 3c showed that CSE1L, BAX, BAK1 were up-expressed in the HCC tissues and BNIP3, CRADD, FAS, SH3GLB1 down-expressed. Hazard ratio (HR) > 1 indicated that the gene was a high-risk gene related to the prognosis of HCC. Figure 4 showed among the 7 intersect Args, BAK1, BAX, CSE1L, and SH3GLB1 were high-risk prognosis-related genes of HCC, BNIP3, CRADD, FAS were low-risk prognosis-related genes.

**Table 2:** Differentially expressed ARGs

Gene	conMean	treatMean	logFC	*p*.value
BAK1	3.6840	7.3115	0.9889	0.0049
BAX	11.4039	22.1529	0.9580	0.0029
BCL2	0.7565	0.6726	−0.1696	0.0416
BCL2L12	4.5640	9.7040	1.0883	0.0013
BNIP3	32.5056	24.7871	−0.3911	0.0283
BNIP3L	11.6700	7.6242	−0.6141	0.0033
CALR	253.8644	564.4069	1.1527	0.0002
CASP7	7.0616	4.9815	−0.5034	0.0037
CASP8	1.9536	2.8375	0.5385	0.0471
CASP9	4.3837	2.3624	−0.8919	0.0160
CFLAR	4.0543	3.2914	−0.3008	0.0178
CRADD	6.0676	3.7604	−0.6902	0.0036
CSE1L	11.4809	20.6950	0.8500	0.0010
FAS	6.2966	3.0988	−1.0229	0.0035
SH3GLB1	8.8173	7.2783	−0.2767	0.0135
TNFRSF1B	24.5535	12.4104	−0.9844	0.0049
PHAP	10.3958	13.9188	0.4210	0.0129
PUMA	1.6533	3.6201	1.1307	0.0034
TRAIL-R4	5.2293	3.1865	−0.7147	0.0156
FASL	0.4797	0.3434	−0.4824	0.0096

**Table 3:** HCC prognostic ARGs

Gene	HR	HR.95 L	HR.95H	*p*.value
AVEN	0.5495	0.3382	0.8928	0.0156
BAK1	2.4836	1.5969	3.8626	0.0001
BAX	1.7013	1.0807	2.6781	0.0217
BMF	1.4375	1.0879	1.8995	0.0107
BNIP3	0.6521	0.4665	0.9113	0.0123
CRADD	0.3687	0.2097	0.6483	0.0005
CSE1L	2.9130	1.8551	4.5741	<0.0001
FAS	0.5185	0.3474	0.7737	0.0013
SH3GLB1	2.3639	1.2372	4.5170	0.0092
NOXA	2.0811	1.3305	3.2553	0.0013

### The results of the construction of the prognostic signature

**Fig. 5:**
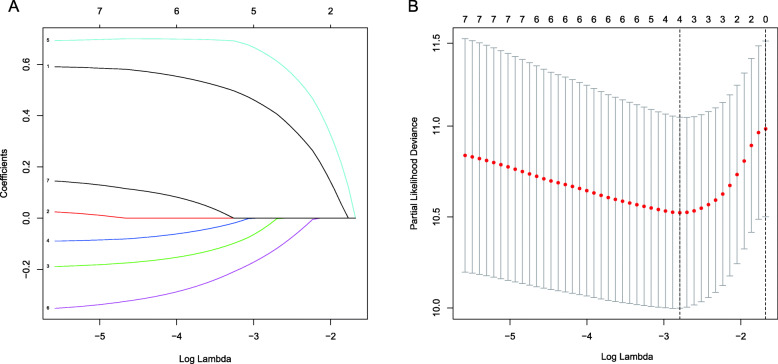
The Construction of a 4-gene prognostic model in the TCGA dataset. **a** Lasso coefficient profiles of the expression of 7 genes. **b** Selection of the penalty parameter (Lambda) in the Lasso model via 10-fold cross-validation

Lasso-Cox regression analysis was applied to establish a prognostic model based on the expression of the 7 genes. According to the penalty parameter (Lambda) in the model, we constructed a prognostic model of HCC patients consisting of 4 genes (Fig. 5). These genes are BAK1, BNIP3, CSE1L, and FAS (Table 4). We then calculated the risk score based on the expression of the 4 genes in the TCGA dataset and corresponding coefficient (coef) (Risk score = e ^(the expression of BAK1*0.4252 + the expression of BNIP3*-0.0237 + the expression of CSE1L*0.6321 + the expression of FAS*-0.1360)^) and divided the patients into high- and low-risk groups according to the median cutoff value of TCGA (risk score < = 3.5614 was low-risk, > 3.5614 was high-risk). The HCC samples of ICGC were also divided into the high- and low-risk groups according to the same median cut-off value.

**Table 4:** The information of the 4 genes used to construct the prognostic signature

Symbol	Description	Coef
BAK1	Bcl-2 homologous antagonist/killer	0.4252
BNIP3	BCL2/adenovirus E1B 19 kDa protein-interacting protein 3	−0.0237
CSE1L	Exportin-2	0.6321
FAS	Tumor necrosis factor receptor superfamily member 6 Precursor	−0.1360

**Table 5:** The clinical correlation analysis

Gene	Age (<=60/> 60) (*p*.value)	Gender (male/female) (*p*.value)	Grade (G1–2/ G3–4) (*p*.value)	Stage (Stage I-II/Stage III-IV) (*p*.value)
BAK1	0.9288(0.3553)	1.6066(0.1146)	−1.1404(0.2559)	−2.1657(0.0343)
BNIP3	−1.8423(0.0683)	−2.4848(0.0172)	0.0997(0.9207)	1.4734(0.1463)
CSE1L	1.3898(0.1675)	0.4319(0.6674)	−1.9311(0.0554)	−4.1353(0.0001)
FAS	−1.7086(0.0904)	−2.4645(0.0168)	1.7182(0.0879)	0.1881(0.8515)
riskScore	1.7350(0.0859)	1.6985(0.0956)	−2.1455(0.0335)	−3.5536(0.0008)

### The results of the validation of the 4-gene prognostic model

**Fig. 6:**
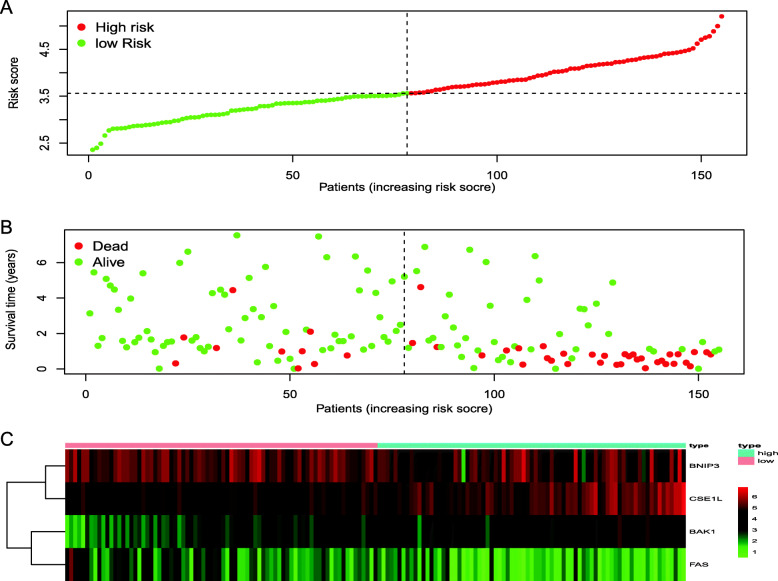
The validation of the reliability of the 4-gene signature by analyzing the risk score and the TCGA dataset. **a** The distribution and the median value of the risk scores. **b** The distributions of OS status, OS and risk score. **c** The expression of four prognosis-related ARGs between high-risk and low-risk group

**Fig. 7:**
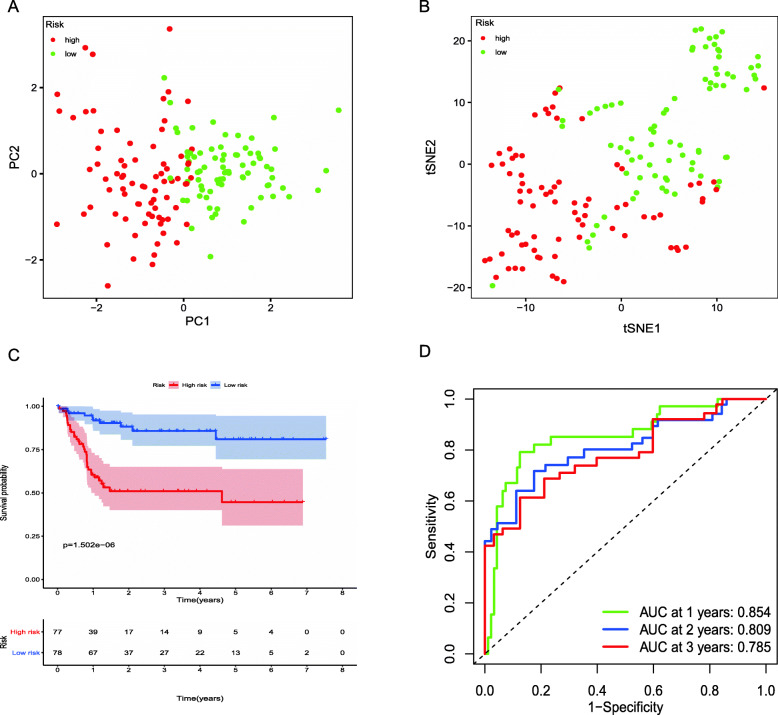
The validation of the reliability of the 4-gene signature using the TCGA dataset. **a** PCA plot. **b** t-SNE analysis. **c** Kaplan-Meier curve showing the OS of patients in the high-risk group and low-risk group. **d** AUC of time-dependent ROC curves verified the prognostic performance of the risk score

**Fig. 8:**
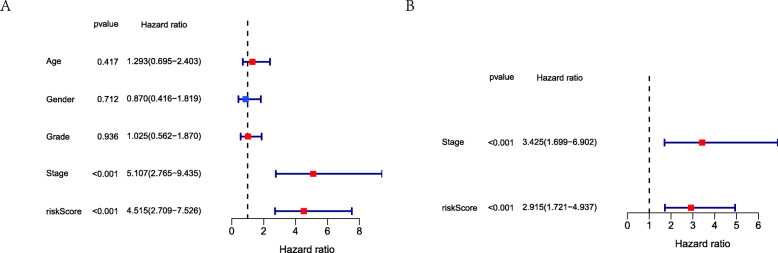
Results of the univariate and multivariate Cox regression analysis regarding OS in the TCGA dataset. **a** The result of univariate Cox regression analysis. **b** The result of multivariate Cox regression analysis

**Fig. 9:**
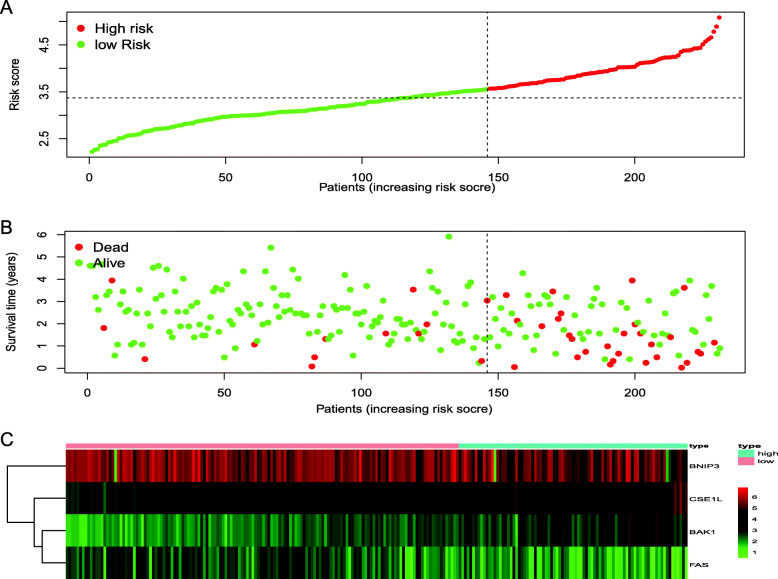
The validation of the reliability of the 4-gene signature by analyzing the risk score and the ICGC dataset. **a** The distribution and the median value of the risk scores. **b** The distributions of OS status, OS and risk score. **c** The expression of four prognosis-related ARGs between high-risk and low-risk group

**Fig. 10:**
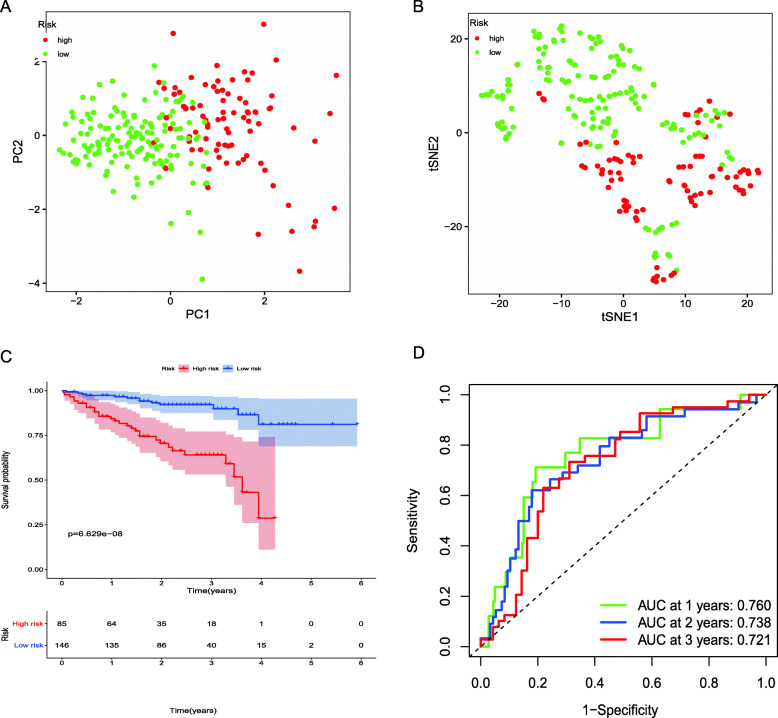
The validation of the reliability of the 4-gene signature using the ICGC dataset. **a** PCA plot. **b** t-SNE analysis. **c** Kaplan-Meier curve showing the OS of patients in the high-risk group and low-risk group. **d** AUC of time-dependent ROC curves verified the prognostic performance of the risk score

**Fig. 11:**
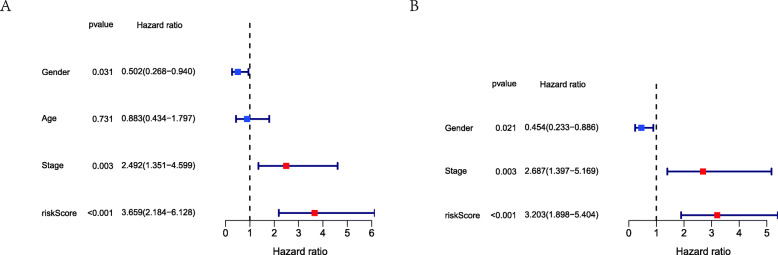
Results of the univariate and multivariate Cox regression analysis regarding OS in the ICGC dataset. **a** The result of univariate Cox regression analysis. **b** The result of multivariate Cox regression analysis

**Fig. 12:**
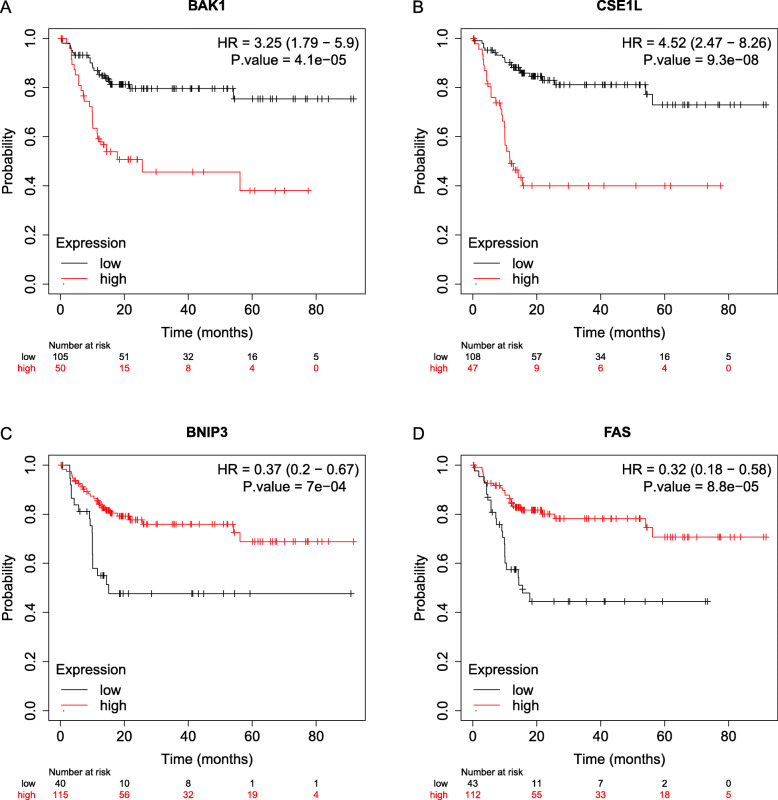
The Kaplan-Meier curves of 4 prognostic model genes. **a**. Kaplan-Meier curve showing the OS of BAK1 in the high-risk group and low-risk group. **b**. Kaplan-Meier curve showing the OS of CSE1L in the high-risk group and low-risk group. **c**. Kaplan-Meier curve showing the OS of BNIP3 in the high-risk group and low-risk group. **d** Kaplan-Meier curve showing the OS of FAS in the high-risk group and low-risk group

**Fig. 13:**
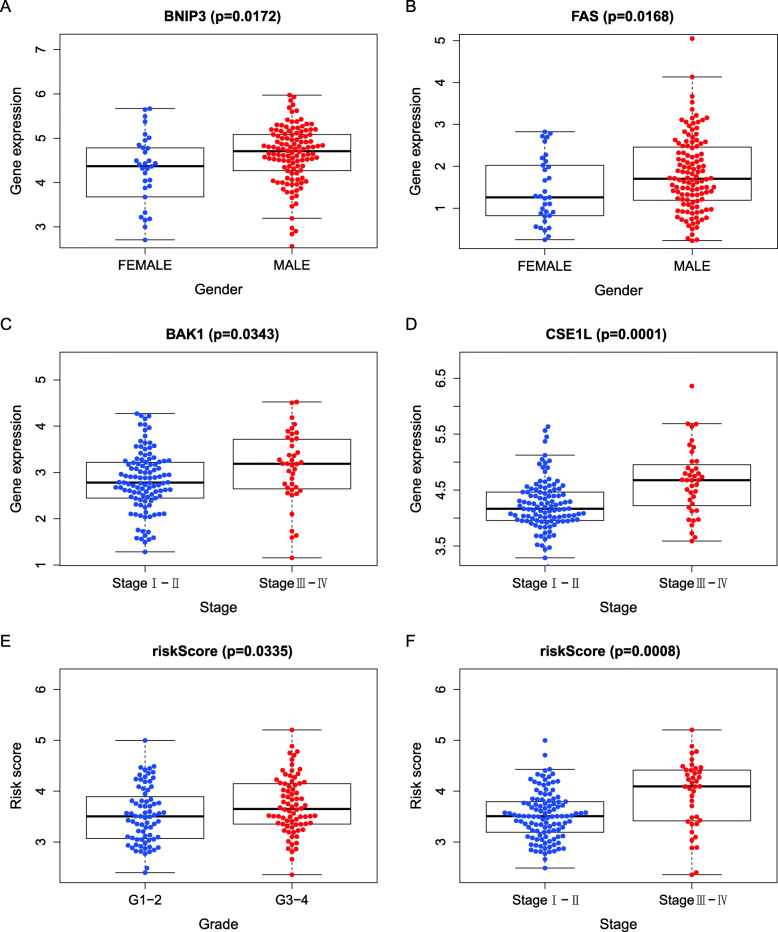
The correlation between gene expression, risk score, and clinical traits. **a** The correlation between the expression of BNIP3 and gender. **b** The correlation between the expression of FAS and gender. **c** The correlation between the expression of BAK1 and HCC stage. **d** The correlation between the expression of CSE1L and HCC stage. **e** The correlation between risk score and HCC grade. **f** The correlation between risk score and HCC stage

We had classified the Asian HCC patients of TCGA database into the high- and low-risk groups according to the median cut-off value (Fig. 6a). Figure 6b showed that in HCC patients of TCGA, the OS of high-risk patients was significantly lower than that of low-risk patients, suggesting the probability of premature death in high-risk patients was higher than that in low-risk patients. What is more, there are significant differences in gene expression. The expression of BAK1, CSE1L in the high-risk group is higher than that in the low-risk group, but BNIP3, FAS was down-regulated in the high-risk group (Fig. 6c). We used PCA analysis and t-SNE analysis for data dimensionality reduction to observe a significant difference between the high- and low-risk groups. The results (Fig. 7a, b) showed that high-risk and low-risk groups of TCGA were a two-way distribution. Kaplan-Meier curve (Fig. 7c) showed that the OS of high-risk patients was significantly lower than that of patients with low risk at the same timing (*p* < 0.001). ROC curves evaluated the predictive performance of the risk score for OS, and the area under the curve (AUC) reached 0.854 at 1 year, 0.809 at 2 years, and 0.785 at 3 years (Fig. 7d). The AUC of the three timings were all higher than 0.700, suggesting that the prognostic model can be regarded as a qualified prognostic biomarker of Asian HCC patients. Then we conducted univariate and multivariate Cox regression analysis to determine whether the risk score model could be used as an independent prognostic factor of OS. In univariate Cox regression analysis, the risk score was significantly associated with the OS of the TCGA HCC patients(*p* < 0.001)(Fig. 8a). After adjusting for other interfering factors, multivariate Cox regression analysis showed that the risk score was still an independent predictor of OS (*p* < 0.001) (Fig. 8b). In order to exclude the contingency, we used the Japanese HCC samples from ICGC database for secondary detection. The HCC samples of the ICGC dataset were also categorized into high- and low-risk groups by the same median value (Fig. 9a). The result showed that most patients with ICGC were at low-risk. Figure 9b showed that OS in the high-risk group of ICGC was also lower than that in the low-risk group. The expression levels of BAK1, CSE1L, BNIP3, and FAS were consistent with those in the TCGA patients (Fig. 9c). The results of PCA analysis and t-SNE analysis showed that the patients in the high-risk group and the low-risk group in the ICGC dataset also showed a two-way distribution (Fig. 10a, b). The Kaplan-Meier curve showed that the OS of HCC patients in the ICGC dataset was lower than that in the low-risk group (*p* < 0.001) (Fig. 10c) at the same timing. Figure 10d displayed the AUC reached 0.760 at 1 year, 0.738 at 2 years, and 0.721 at 3 years. The AUC of the three-time points were all higher than 0.700 as well. We performed univariate and multivariate Cox regression analysis to determine whether the risk score model was also an independent prognostic factor of OS in HCC patients of ICGC. Through the analysis, we found that risk score could be regarded as an independent prognostic factor of the OS of IGCG HCC patients as well (*p* < 0.001) (Fig. 11a, b). Moreover, to verify the relationship between the prognosis model and different clinical traits, we divided the Japanese HCC samples from ICGC database into a high-risk group and low-risk group according to the risk value and drew the Kaplan-Meier curves based on the gender, age, and HCC stage (Fig. 12). The results showed that the HCC prognostic model could also be used as the prognosis of HCC with different age, gender, and stage. Meanwhile, because only used the Japanese HCC dataset for validation to reduce the regional influence of HCC disease, the data of Kaplan Meier plotter database was used to verify again. We selected HCC as the research disease and 155 Asian patients as the research objective. The expression of 4 genes (BAK1, BNIP3, CSE1L, FAS) used to construct the predictive model were selected, and the Kaplan-Meier curves were drawn, respectively (Fig. 13). The results suggested that the prognostic model can be seen as an effective prognostic factor for Asian HCC patients.

### The results of correlation analysis with clinical traits

**Fig. 14:**
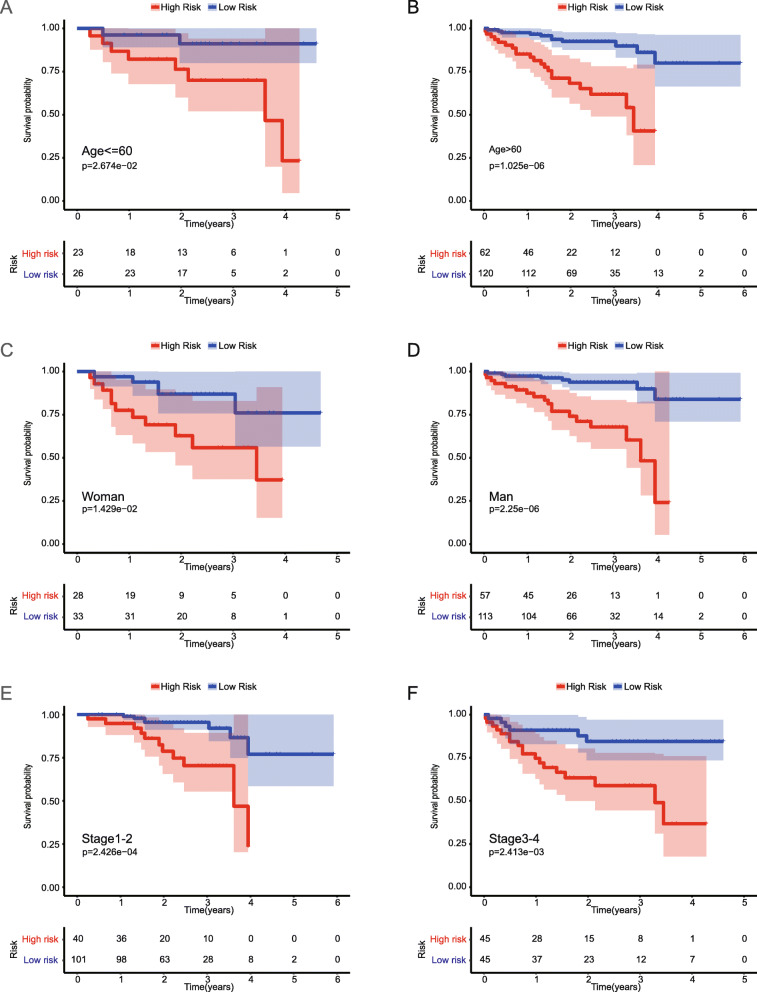
The Kaplan-Meier curves of clinical traits. **a** Showing the OS of younger (age < =60) in the high-risk group and low-risk group. **b** Showing the OS of older (age > 60) in the high-risk group and low-risk group. **c** Showing the OS of women in the high-risk group and low-risk group. **d** Showing the OS of men in the high-risk group and low-risk group. **e** Showing the OS of stageI-II in the high-risk group and low-risk group. **f** Showing the OS of stageIII-IV in the high-risk group and low-risk group

The TCGA-BC-A10W and TCGA-ZP-A9CZ cases without HCC stage information were deleted. T-test was used to determine whether the expression of the genes and the risk score were associated with the clinical characteristics of HCC patients. Some clinical features of HCC, including age, sex, grade, and stage, were assessed for their probable correlation with BAK1, BNIP3, CSE1L, FAS, and risk score, as shown in Table 5. We selected the correlation, which satisfies *p* < 0.05 to draw the box plots. The results showed that the expression of BNIP3 and FAS were significantly different between male and female (Fig. 13a). BAK1 and CSE1L were all significantly differentially expressed between Stage I-II and Stage III-IV patients (Fig. 13b-d). The values of risk score were significantly different between the Grade 1–2 and Grade 3–4 (*p* < 0.05) (Fig. 13e). The values of risk score were different between Stage I-II and Stage III-IV, as well. (*p*<0.001) (Fig. 14f). Correlation analysis of clinical traits showed that risk score was near related to the grade and stage of HCC. We also used the clinical character data of Japanese HCC patients in the ICGC database. The relationship between different clinical traits (Age, Gender, Stage) and OS was showed by Kaplan-Meier curves, respectively. The results (Fig. 14) showed that the prognosis of HCC was Significant different with age, gender, and cancer stage in both high-risk and low-risk groups divided according to the median risk value of the prognostic model (*P* < 0.05). The result further demonstrated the prognostic model we constructed for Asian HCC patients is reliable.

## Discussion

Large geographic disparities in incidence and mortality of HCC exist. Asia has the highest incidence of HCC in the world because of the presence of multiple risk factors, such as hepatitis B and the contamination of aflatoxin [81]. Another risk factor for HCC, chronic hepatitis C infection, in Asia is most significant in Japan, the only Asian country with more hepatitis C virus (HCV) than hepatitis B virus (HBV)-related hepatocellular carcinoma. To eliminate the cells infected by viruses, the body will activate apoptosis, but to reduce the death of cancer cells, HCC cells will inhibit apoptosis. The balance of survival and apoptosis are closely related to the progression of HCC [12]. The etiology of HCC in Asia is quite different from that in Europe, the US, and the balance of survival and apoptosis is crucial for the progression of HCC. Therefore, it is necessary to study the cases of Asian HCC patients and find a useful Args related prognostic model based on the genes of Asian populations.

In the study, we systematically studied the expression of 56 Args and the effect of prognosis. And we constructed a new prognostic model based on apoptosis-related DEGs and verified it by the data of TCGA and ICGC databases. The HCC prognostic model consists of four Args (BAK1, BNIP3, CSE1L, and FAS). Pro-Apoptotic Protein BAK (BAK1) is a pro-apoptotic protein [21], which plays a vital role in the process of mitochondrial apoptosis. When receiving an apoptosis signal, BAK1 can change the permeability of mitochondrial outer membrane (MOM), release apoptotic factors, and activate effector caspases to realize apoptosis [82, 83]. BNIP3, a pro-apoptotic member of the Bcl-2 family of apoptotic proteins [84], can overcome the inhabitation of apoptosis caused by BCL2. However, some studies have found that BNIP3 has an inhibitory effect on cancer [85]. BNIP3 can delay the progression of primary breast cancer by preventing the accumulation of dysfunctional mitochondria and reducing the resulting excess reactive oxygen species (ROS) [86]. CSE1L, the cellular apoptosis susceptibility protein, is highly expressed in various cancers [87]. It has been found that CSE1L plays an essential role in regulating apoptosis induced by chemotherapeutic drugs [88]. CSE1L can inhibit paclitaxel-induced apoptosis by affecting G2/M phase cell cycle arrest and microtubule aster formation induced by paclitaxel [88, 89]. The Fas-antigen is a cell surface receptor that transduces apoptotic signals into cells [90]. Fas/FasL signaling pathway will promote cell apoptosis.

In the study, we found that BAK1, CSE1L, BNIP3, and Fas were all related to the prognosis of HCC, but the expression changes were different. BAK1 and CSE1L were up-regulated in HCC tissues, while BNIP3 and Fas were down-regulated. We recognized that it is not clear whether these genes affect the prognosis of HCC patients mainly by affecting cancer cell apoptosis because these genes affect the progress of HCC in many ways, not only apoptosis. Therefore, further studies on these four genes are needed.

## Conclusion

We successfully constructed a novel apoptosis gene-related prognostic model of accurately predicting the prognosis of Asian HCC patients, with higher risk scores demonstrating adverse prognosis. Kaplan-Meier curve, PCA analysis, t-SNE analysis, risk score curve, ROC curve, and the data of ICGC were used to verify the reliability of the model. We believed this model could act as a useful independent prognostic predictor for Asian HCC patients.

## Supplementary Information

**Additional file 1.**

## Data Availability

All the data used in the study are obtained from the TCGA database (https://portal.gdc.cancer.gov/), ICGC database (https://dcc.icgc.org/), Deathbase database (http://deathbase.org/), and Kaplan-Meier plotter database (http://kmplot.com/analysis/) which are opening and available to all. All datasets generated for this study are included in the manuscript and the supplementary files.

## Abbreviations

HCC: Hepatocellular Carcinoma
Args: Apoptosis-related genes
DEGs: Differential expressed genes
PRGs: Prognostic related genes
TCGA: The Cancer Genome Atlas
ICGC: International Cancer Genome Consortium
NCBI: National Center for Biotechnology Information
Lasso: Least absolute shrinkage and selection operator
COX: Proportional hazards model
CAD: Caspase-activated deoxyribonuclease
PCA: Principal Component Analysis
t-SNE: t-distributed Stochastic Neighbor Embedding
ROC: Receiver operating characteristic
AUC: Area under curve
OS: Overall survival
GO: Gene ontology
KEGG: Kyoto Encyclopedia of Genes and Genomes
HR: Hazard ratio
coef: Corresponding coefficient
HBV: Hepatitis B virus
HCV: Hepatitis C virus
MOM: Mitochondrial outer membrane
ROS: Reactive oxygen species
